# Challenges in nanomedicine clinical translation

**DOI:** 10.1007/s13346-020-00740-5

**Published:** 2020-03-12

**Authors:** Josbert M. Metselaar, Twan Lammers

**Affiliations:** 1 Institute for Experimental Molecular Imaging, RWTH Aachen University Clinic, 52074 Aachen, Germany; 2 Institute for Biomedical Engineering, Faculty of Medicine, RWTH Aachen University, 52074 Aachen, Germany

**Keywords:** Nanomedicine, Drug delivery, Clinical translation, Pharmaceutical development

## Abstract

New nanomedicine formulations and novel applications of nanomedicinal drugs are reported on an almost daily basis. While academic progress and societal promise continue to shoot for the stars, industrial acceptance and clinical translation are being looked at increasingly critically. We here discuss five key challenges that need to be considered when aiming to promote the clinical translation of nanomedicines. We take the perspective of the end-stage users and consequently address the developmental path in a top-down manner. We start off by addressing central and more general issues related to practical and clinical feasibility, followed by more specific preclinical, clinical, and pharmaceutical aspects that nanomedicinal product development entails. We believe that being more aware of the end user’s perspective already early on in the nanomedicine development path will help to better oversee the efforts and investments needed, and to take optimally informed decisions with regard to market opportunities, target disease indication, clinical trial design, therapeutic endpoints, preclinical models, and formulation specifications. Critical reflections on and careful route planning in nanomedicine translation will help to promote the success of nanomedicinal drugs.

**Figure:**
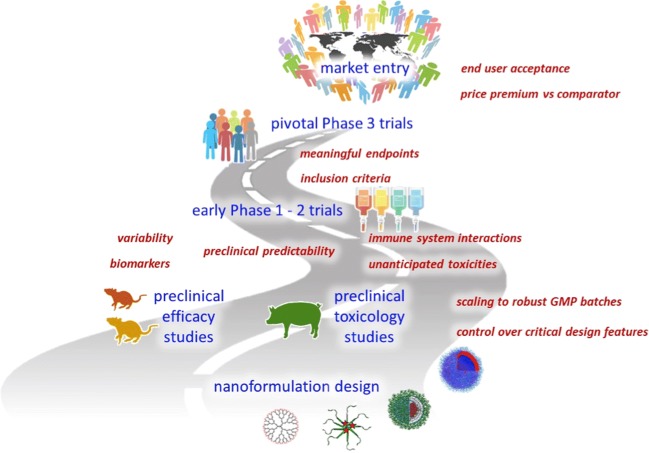
Graphical abstract

Graphical abstract

## Introduction

Nanomedicines are a highly diverse group of drug products. They encompass polymer-drug conjugates, polymer-protein conjugates, protein-based nanoparticles, polymeric micelles, inorganic nanoparticles, and a range of lipid-based nanoparticulate drugs, of which PEGylated liposomes are the prime example [1–3]. Many thousands of different nanomedicine formulations have been designed and evaluated over the years, for various different types of diseases. Approximately fifty of these formulations are currently approved for clinical use, and there are several hundreds of trials ongoing in which nanomedicines are being evaluated in patients [4, 5].

With the exception of nucleic acid–based nanotherapeutics, such as the lipid-based small-interfering RNA formulation patisiran, nanomedicinal drug products are typically reformulations of active pharmaceutical ingredients (API) which have already been approved for clinical use. This makes sense, as improving the in vivo performance, efficacy, or safety of an established API is a less venturesome enterprise as compared with developing a completely new chemical entity (NCE), which has unknown pharmacological and toxicological behavior in human beings. Another advantage of reformulating existing actives is that nanomedicine products of established API may—if proven more efficient and/or less toxic in clinical trials—simply take over the therapeutic positioning which the actives already had in the treatment algorithm, while completely new nano-drug products bear the risk of having to enter last in line. Furthermore, with the pharmacology sorted out and the array of diseases amenable to therapy with the API itself known, the nanomedicine formulation of the API may quickly realize its upside potential once market allowance for a first indication has been granted.

The formulation complexity and the extra care needed in successful nanomedicine upscaling and product development are additional issues to keep in mind. Eventually, if one is to render nanomedicine development viable and commercially feasible, they must all be offset by the therapeutic value of allegedly improved in vivo performance. Indeed, while the academic community justifiably tends to shy away from commercial viability questions in pursue of its research goals, it is clear that with an average clinical drug development program costing around 350 million USD (and typically exceeding 1 billion USD when including marketing costs), investments by venture capital firms and/or large pharmaceutical industries are inevitable. Hence, in our opinion, the crucial question of initial risk-of-investment versus eventual return-on-investment should already be asked and addressed at a very early stage.

**Fig. 1:**
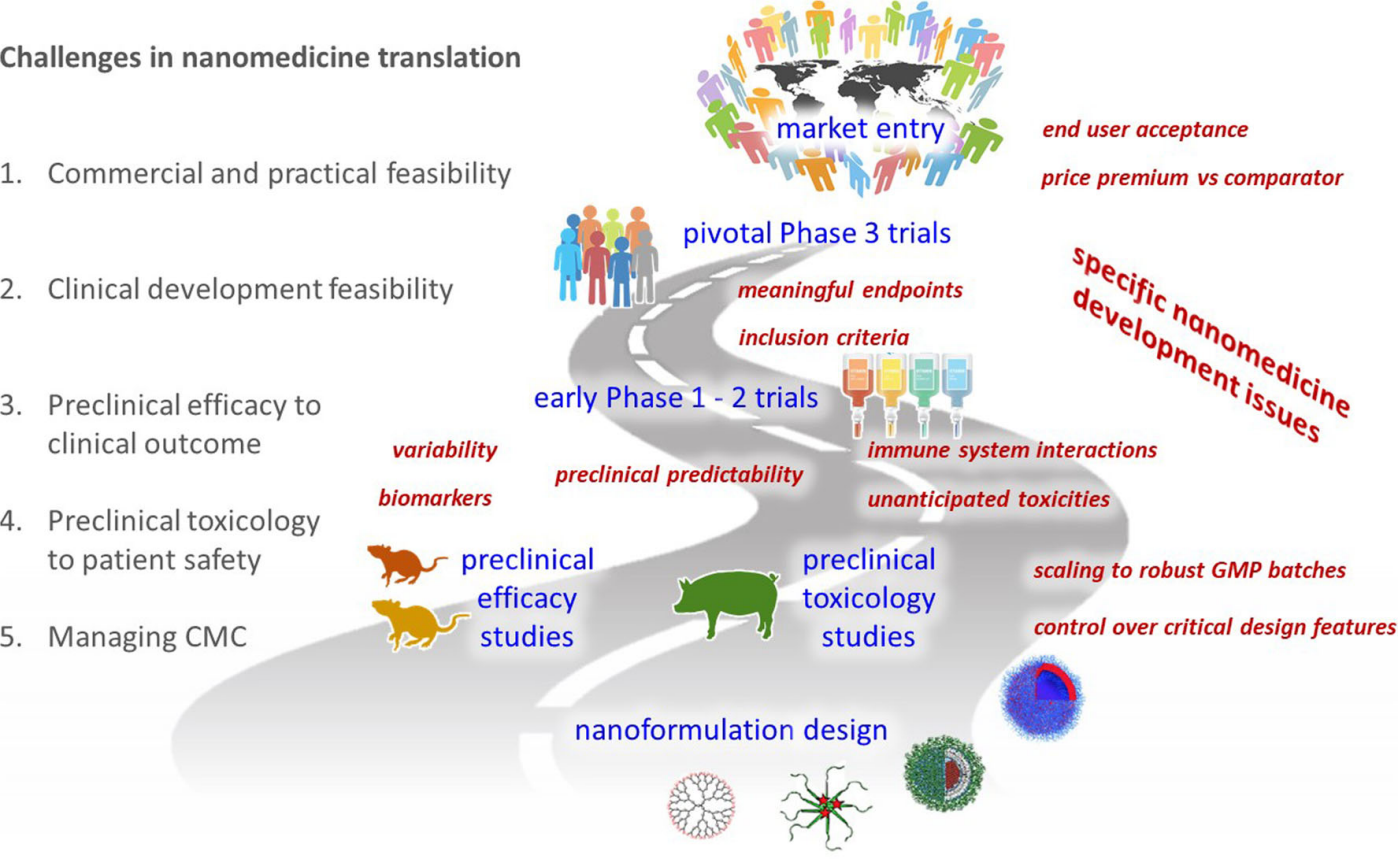
Challenges in nanomedicine translation. Five key challenges of nanomedicinal product development are depicted top-down, from the vantage point of the end user. Practical and clinical development feasibilities come first, followed by preclinical and pharmaceutical aspects of nanomedicine research and development. This way of route planning allows one to identify—right from the start—where and which specific issues can be encountered along the way

To improve the chances of academic nanomedicine projects to reach the clinic and be taken forward towards product development, we advocate for a more proactive attitude towards singling out and endorsing those projects which really have clinical and commercial potential. Here, we therefore discuss key developmental challenges, starting with the broader commercial feasibility questions, then touching upon core clinical issues, and finally addressing preclinical and pharmaceutical aspects we think should be considered before clinical translation is pursued. These challenges can be used as a scoring board in the evaluation of clinical potential, as well as a means to develop adequate risk-mitigation strategies, which is something that investors and commercial parties typically already want to see at relatively early developmental stages. Acknowledging that these five challenges pertain to all drug development programs, we have put particular emphasis on how we feel they specifically apply to nanomedicine formulations (Fig. 1).

## Commercial and practical feasibility

The first challenge to consider before any nanomedicine is taken into development relates to commercial and practical feasibility in view of its primary target indication. Here, both the potential for improved patient benefit and the size of the eventual patient population are important. Improved patient benefit can come with increased therapeutic efficacy, less toxicity or simply by the nanomedicine formulation requiring less frequent dosing or enabling a more convenient administration route than the comparator product (thus promoting patient compliance). The clearer the benefit is, the easier a significant price premium can be justified once the product enters the market. This in combination with the number of patients who will eventually use it determines the potential market and sales for the product, and it will entice investors and commercial parties to step in. However, if the comparator product is a simple cheap tablet and its API is now turned into an advanced nanomedicine specialty product, the first question that comes to mind is whether physicians or patients are willing to change from an easy daily oral routine medication to a parenteral treatment, which is typically given in an outpatient clinic and which may be less frequently needed, but comes at a multiple-fold increased total treatment cost. Taking the above into account, chances are slim that the several hundreds to even thousands of dollars per treatment needed to make a nanomedicine product commercially viable are ever going to be paid for, unless an unambiguous narrative can be told about the potential for a better outcome in long-term disease progression (better efficacy) or for a clear reduction of costly adverse events (better safety). We consider it important to ponder these end-stage marketability questions all the way at the beginning, before a nanomedicine development program is embarked upon.

## Clinical development feasibility

Better efficacy or safety, as defined in the terms above, are very difficult to prove and require large and lengthy clinical trials, which are capital-intensive. This brings us to the second challenge, i.e., clinical development feasibility, where we are specifically looking at issues that come with proper clinical study design. To ensure that a new nanomedicine drug product eventually shows sufficient clinical potential in a pivotal trial, endpoints must be chosen that adequately reflect the anticipated improved patient benefit, and that both society (government bodies) and payors (insurances) are willing to pay for. Showing superiority over standard-of-care seems straightforward, but highly coveted and more clinically relevant outcomes, such as improved disease modulation, better long-term remission, and longer delay of disease progression or avoidance of disability, require long studies and large trial populations. If better safety is aimed for—and this is something payors are also willing to pay for, e.g., the reduction of costly fractures or deadly infections—then, this must be shown against equal efficacy, which typically requires a very large non-inferiority trial design. After all, trial population size (and patient stratification, which is closely related (see below)) is an important point of attention and in itself very much determined by the expected variation in endpoint results, highlighting the importance of carefully assessing this beforehand in phase 2 studies (and to take measures to reduce this). It may seem clever to limit patient variability by tightening the inclusion criteria for a trial, but this comes with two important downfalls: a smaller patient pool to recruit from, often meaning slower enrollment; and the fact that tight inclusion criteria can translate into a restriction of the total amenable patient population for which the product can be labeled once it has made it to the market. Among the options to deal with this is the implementation of biomarkers predictive of therapeutic responses, which lowers the impact of variability (but not the issue of slow enrollment). Many new drug products that now appear on the market come with their own biomarker, and typically, these biomarkers are identified and evaluated early on in the developmental process. In this regard, one could even argue that biomarkers should already be identified before clinical development is commenced, and there is no reason why this—as for molecularly targeted therapeutic and monoclonal antibodies—should not also hold true for nanomedicinal drugs [6–8].

## Translating preclinical efficacy to clinical outcome

The third challenge relates to the translation of preclinical efficacy to clinical outcome. For nanomedicines, issues regarding response rate variability and therapeutic efficacy prediction in patients may all be a bit more complicated than for other drug products, particularly for molecularly targeted therapeutics [9]. Most of us are nowadays already quite careful with assigning too much importance to positive preclinical study outcomes and there are myriads of examples of drugs that in patients do not live up to expectations based on raving animal study results, notoriously so in the field of oncology. The observation that nanomedicines seem to be more prone to lack of predictability of patient benefit as compared with conventional drugs and molecularly targeted therapeutics may be due to the critical dependence of nanomedicine efficacy on pharmacokinetics, tissue distribution, target site accumulation and penetration, and drug release at the target site (and ideally in the target cell), which are all specific in vivo nanoparticulate performance aspects and which are very different in animal models versus in human patients. Lessons learnt in anticancer nanomedicine development have taught us the importance of tumor vascularization, stroma, and especially macrophage population in nanoparticle target localization and drug release, and these tissue morphology aspects typically vary dramatically inside a tumor, across tumors in a patient and even more so among (different cancerous lesions in) different patients [7–9]. This becomes all the more poignant when we realize that thus far, the (cancer) nanomedicine field has seen relatively little progress in the development of biomarkers and companion diagnostics. To properly translate good preclinical efficacy to good clinical outcome, we therefore have to make sure that we start developing tools and technologies to assess and address variability in nanomedicine performance in patients.

## Bridging preclinical toxicology to patient safety

The fourth challenge relates to closing the gap between preclinical toxicology studies and ensuring safety in patients. Nanomedicines can pose safety issues at three different levels (beyond the intrinsic toxicity of the formulated API itself). Firstly, when delivered via a nanoparticle, the biodistribution of drug molecules often dramatically changes, and the resulting uptake in certain organs may lead to local overexposure. The propensity of nanoparticles to accumulate in lymphoid organs is well-known, as is the preferential accumulation in the kidneys of some polymer-bound drugs. Secondly, unexpected nanomedicine-related toxicity can result from excipients not yet proven safe in humans. To address potential safety issues at these two levels, it is advisable to use early preclinical pharmacokinetic and biodistribution studies, and to assess organ drug exposure and toxicity by doing extensive histopathology as well as established clinical chemistry protocols, taking drug-free nanocarriers in different doses along as key controls. The third level at which nanomedicine-related safety issues can occur relates to immunological responses, which are difficult to predict based on studies in small laboratory animals. These, e.g., refer to hypersensitivity reactions, which are only seen in a relatively small percentage of humans upon nanomedicine administration, but which can be quite severe and sometimes even life-threatening. The activation of the complement cascade plays a major role in immunological side effects, and also specific nanoparticle-blood cell interactions have been reported to contribute to this. To address these issues, in vitro complement binding and cell interaction assays can be employed, and also preclinical safety studies in larger animals, notably in pigs, are advisable [10].

## Chemistry, manufacturing, and control

The fifth and final challenge that needs to be addressed is proper management of chemistry, manufacturing, and quality control (CMC) of nanomedicinal drugs. The main difference with conventional drug products is that the in vivo performance of nanomedicines, such as biodistribution, target accumulation, and drug availability at pathological and non-pathological sites—on which their efficacy as well as their safety critically depends—is a direct consequence of the physicochemical properties of the nanoparticle that contains the drug. While quality control must be tight for any medicinal product, nanomedicine or not, the criticality of particle size, surface morphology, drug loading, release, and quite likely a few others involves a range of quality control assays on top of the standard array of quality checks. It is absolutely paramount that critical quality attributes, such as particle size, size distribution, charge and morphology, and drug encapsulation and release, are considered very early on in formulation design, and that narrow specifications are defined within which the formulations show optimal performance. Manufacturing should be robust and scalable and performed under stringent adherence to good manufacturing practice (GMP) guidelines. In addition, it should preferably be done according to quality-by-design (QbD) principles, meaning that adequate in-process controls should be implemented that enable the monitoring of key quality attributes during compounding, giving the manufacturer time to adjust critical process parameters, such as temperature and pressure, to safely steer the final formulation within the set specifications. With this in mind, it makes sense to carefully assess composition, excipients, and key quality attributes (including quality control assays) as early on as possible in the design of a nanomedicine drug, preferably even before preclinical testing in animal models. Scalable manufacturing methods are typically developed later; it is nonetheless advisable to be aware of potential caveats, such as sterility and manufacturing-related impurities, as early as possible in the whole process, to try to avoid a costly reformulation process, which would in the worst case require the entire preclinical data package to be revisited. Consequently, carefully and comprehensively considering chemistry, manufacturing, and control aspects as soon as possible in nanomedicine development crucially contributes to translational success [11].

## Concluding remarks

Nanomedicines keep on targeting new drugs and new disease indications while gaining further momentum in the clinical arena. We—the nanomedicine research community—should not only be watching from our rather safe academic vantage point but should really try to envisage the implementation in clinical practice with every research effort we undertake. We believe that by creating stronger awareness of the end user’s perspective, a clearer picture arises of the overall efforts and investments needed, right at the stage of formulation design and laboratory bench experimentation. When the market opportunity and therapeutic positioning have been pondered and the clinical trial design including its therapeutic endpoints carefully considered, then, one can take informed decisions not only on preclinical experimental setup but also on formulation specifications and even manufacturing methods. This way of careful route planning and navigation through nanomedicine clinical translation is needed to help investigational nanomedicinal drug products eventually deliver on their promise of increased patient benefit.
